# Editorial: Maternal-foetal crosstalk impacts on offspring development

**DOI:** 10.3389/fcell.2023.1133159

**Published:** 2023-01-17

**Authors:** P. Ybot-Gonzalez, N. D. E. Greene, A. J. Copp, D. J. Henderson, V. Massa

**Affiliations:** ^1^ Neurodevelopment and Neuropaediatric Diseases Research Group, Institute of Biomedicine of Seville, IBIS/HUVR/CSIC/US, Seville, Spain; ^2^ Developmental Biology and Cancer Department, UCL GOS Institute of Child Health, London, United Kingdom; ^3^ Bioscience Institute, Faculty of Biomedical Sciences, Newcastle University, Centre for Life, Newcastle upon Tyne, United Kingdom; ^4^ Department of Health Sciences, Università Degli Studi di Milano, Milan, Italy; ^5^ “Aldo Ravelli” Center for Neurotechnology and Experimental Brain Therapeutics, Università Degli Studi di Milano, Milan, Italy

**Keywords:** maternal effect, maternal supplementation, risk factors for congenital diseases, teratogen exposure, embryopathies

Embryogenesis involves complex processes that are tightly regulated by different signalling pathways, the activity of which can be altered by genetic and environmental factors. Lessons learned from nature show us that the environment, condition and/or genotype of the mother can modulate the phenotype of her offspring, in some cases reversing the developmental instructions conferred by the offspring’s genotype. This is what is known as the “maternal effect,” which gives plasticity to the phenotype of her offspring to adapt to different environmental situations. Although maternal effect could have a beneficial connotation aimed to increase the fitness of the offspring, it has to be considered that it may be detrimental to the wellbeing of the embryo. In humans, among the environmental factors that affect the intrauterine environment during embryogenesis, we must consider maternal health, medications, socioeconomic status, lifestyle and genotype. Some of these environmental factors could have their potentially adverse effects by altering the expression of genes involved in the signalling and metabolic pathways implicated in embryogenesis. For instance, maternal hyperglycaemia, one of the most studied chronic maternal diseases in relation to embryopathies, affects the expression of the components of a wide range of signalling pathways, suggesting that this interference could be the basis of diabetic embryopathies. Understanding the mechanisms underlying diabetic embryopathy demands an understanding of the expression and localisation of relevant transporters that mediate nutrient uptake by the developing embryos (Kappen et al.). Among the risk factors for the development of type 2 diabetes, the most common form of diabetes (90% of cases), include overweight and obesity, which are becoming one of the most important public health challenges worldwide. Obesity among women of childbearing age is also on the rise and it is now widely accepted that maternal nutritional status in early life can influence the long-term health of offspring. Thus, it is crucial to understand how parental obesity alters the metabolic programmes of offspring by affecting the development of gametes, placenta, adipose tissue, pancreatic β cells, and hypothalamic circuits that control feeding (Cechinel et al.). Along the same lines, maternal obesity has also been reported to modulate the microbiota, affecting nutrient absorption and vitamin synthesis, with direct repercussions on embryonic development. Therefore, obesity due to poor maternal nutrition during pregnancy has a negative impact on neurodevelopmental outcomes and plays an important role in the induction of mental disorders such as depression and schizophrenia in the offspring (Jantsch et al.). Furthermore, there is evidence suggesting an association between prenatal exposure to respiratory infections, such as SARS-CoV-2 and influenza, with an increased risk of neurodevelopmental disorders in offspring, like schizophrenia, bipolar disorder and autism. The current pandemic situation of COVID-19 seems to be a unique situation for further research in this field.

Other maternal factor to be taken into account in the development of the embryo is placentation as it brings the foetal and maternal circulations closer together to facilitate an efficient exchange of gases, nutrients and wastes. The normal development of the placenta, and thus of the embryo, depends on the successful establishment of early placental angiogenesis. Thus, syncytin, an endogenous retroviral envelope protein, plays an essential role in placental angiogenesis and is associated with the pathophysiology of placental-related diseases such as foetal growth restriction (Wang et al.).

Another relevant environmental factor is the use of pharmacological treatments in women of childbearing age, which represents a unique therapeutic problem where, while the mother may benefit from a given treatment, it may have an adverse effect on the foetus. It is important to note that since almost all drugs in the maternal circulation cross the placenta, and xenobiotic metabolism varies greatly from embryo to foetus and throughout the animal’s life, a variation in the activity of metabolizing enzymes in the embryo could produce teratogenic metabolites. In this area, the huge repercussions caused by the teratogenic effects of thalidomide come to mind. Other medicines with a proven teratogenic effect are antiepileptic drugs, with a 2 to 4 times higher risk of inducing congenital malformation, as well as adverse neurobehavioral outcomes in adult offspring with no other apparent malformations during pregnancy. To this end, Steele et al. showed that levels of Valproate metabolites vary in susceptible and non-susceptible (to valproate-induced neural tube defects; NTD) strains of mice. Interestingly, correcting for low levels of one specific metabolite in the NTD-susceptible strain was able to reduce the incidence of NTDs, suggesting that better understanding variation in drug metabolism at a population level is a step towards reducing teratogenicity. Moreover, the negative role of maternal alcohol consumption is known but it still represents a major risk in many countries with a 19.6% of reported pregnant women using alcohol during first trimester in USA alone. The neurobehavioral effects on the offspring vary in severity but represent a leading cause of preventable intellectual disability worldwide. Nevertheless, the neuropathology and multigenerational effects are still yet to be fully elucidated (Bottom et al.).

Despise these cautionary tales, it is precisely through the mother that effective strategies can be designed for the prevention of developmental diseases by altering the foetal environment and, in turn, modulating the phenotype of the foetus. Maternal supplementation with folic acid and inositol has been shown to be effective in reducing the incidence of neural tube defects, and diminishing maternal homocysteine concentrations through folate and vitamin B12 supplements can have a beneficial effect on embryonic development, as well as contributing to improved maternal health (D’Souza and Glazier). A genetically determined alteration in folate one-carbon metabolism also has potential for multi-generational effects on development, as exemplified by conceptus alignment and spacing (Wilkinson et al.).

Thus Maternal nutrition during pregnancy is an increasingly interesting Research Topic, as it can have an impact both on mothers and on the development of their offspring.

